# The effectiveness of liposomal bupivacaine in ultrasound‐guided abdominal wall blocks after open abdominal surgery: A systematic review

**DOI:** 10.1111/papr.70016

**Published:** 2025-02-19

**Authors:** Maya S. Vereen, Vincent J. Bidault, Elise Krabbendam, Sanne E. Hoeks, Robert Jan Stolker, Maaike Dirckx

**Affiliations:** ^1^ Department of Anaesthesia Erasmus Medical Centre Rotterdam The Netherlands; ^2^ Medical Library Erasmus Medical Centre Rotterdam The Netherlands

**Keywords:** analgesics, anesthesia, anesthetics, local, opioid, postoperative pain, regional

## Abstract

**Background and Objective:**

Thoracic epidural analgesia has traditionally been used for pain management after open abdominal surgery, but its use has declined. The quest for efficient alternatives has resulted in the increasing use of regional techniques. These can be applied as single‐shot or continuous blocks using catheters. Long‐acting liposomal bupivacaine could preclude the use of catheters. This review aimed to evaluate the effectiveness of ultrasound‐guided abdominal wall blocks with liposomal bupivacaine for open abdominal surgery.

**Databases and Data Treatment:**

Medline ALL, Embase, Web of Science Core Collection, Cochrane Central Register of Controlled Trials, and Google Scholar were systematically searched. Screening, data extraction, and quality assessment were done by two independent researchers. Inclusion criteria were (1) liposomal bupivacaine in ultrasound‐guided abdominal wall blocks for open abdominal surgery, (2) outcome of pain and/or opioid consumption, (3) patients >18 years, and (4) reports published in English.

**Results:**

Of the 1277 studies found, 22 met the inclusion criteria. The Cochrane Risk of Bias (Version 2) tool was used to assess randomized controlled trials. Studies were grouped for clarity. Transversus abdominis plane (TAP) blocks were mostly investigated. Data were heterogenic regarding types of surgery, approach to block placement, anesthetic solution injected, and use of intrathecal morphine (ITM).

**Conclusions:**

Patients undergoing cesarean section with neuraxial anesthesia and intrathecal morphine benefit from TAP blocks with liposomal bupivacaine, demonstrating reduced opioid consumption and comparable pain. Evidence for other open abdominal surgeries was inconclusive. Abdominal wall blocks with liposomal bupivacaine could be a viable alternative when epidural analgesia is contraindicated.

## INTRODUCTION

Optimal perioperative pain management after open abdominal surgery is essential as it can reduce the surgical stress response, protect the myocardium, and result in earlier mobilization.^1^, ^2^ Severe acute postoperative pain is a risk factor for the development of chronic postsurgical pain, with possible long‐term implications.^3^

Thoracic epidural analgesia (TEA) has been the traditional approach for optimal pain management after open abdominal surgery, treating both visceral and somatic components of pain.^4^ Lumbar epidural analgesia is also a possibility for pelvic and obstetric surgery.^5^ The use of epidural analgesia has, however, decreased over the past decades due to the development of minimally invasive surgical techniques, emphasis on early ambulation, the potential for postoperative hypotension, and the increased use of anticoagulation therapies.^6^

The emergence of Direct Oral Anticoagulants (DOACs), newer anticoagulant therapies, has further limited the use of neuraxial analgesia, especially for emergency surgery, because they need to be stopped 24–72 h beforehand.^7^ This has resulted in a search for effective alternatives for epidural analgesia, such as regional techniques including transversus abdominis plane (TAP) and rectus sheath (RS) blocks.^8^, ^9^ The advantages include somatic analgesia only, without hypotension and risk to the neuraxis. Initially, abdominal wall blocks were placed using landmarks, while the current standard is ultrasound guidance.^10^ This has facilitated the development of existing blocks, to blocks possibly treating visceral pain, such as erector spinae plane (ESP) and quadratus lumborum (QL) blocks (4; 11).

All abdominal wall blocks can be applied as single‐shot or continuous techniques by insertion of catheters.^10^ Catheters can be attached to infusion or elastomeric pumps for constant infusion of local anesthetic, or can be manually injected intermittently with a bolus of a local anesthetic. Catheters can migrate or dislodge, resulting in suboptimal or no analgesia, and could impede mobilization if attached to infusion pumps.^11^

Liposomal bupivacaine (LB) consists of multivesicular liposomes encapsulating bupivacaine within aqueous chambers using DepoFoam® technology (Pacira Pharmaceuticals Inc.).^12^ It produces reliable plasma concentrations of bupivacaine for up to 72 h after injection.^13^, ^14^ The expected prolonged duration of analgesia is promising and might preclude the necessity for catheters. Supporting data has, however, demonstrated conflicting results.^15^ A review addressing LB in TAP blocks for all types of surgical procedures, including different techniques of TAP block placement (ultrasound‐guided, laparoscopic‐assisted and under direct vision) found inconclusive evidence for prolonged analgesia with LB.^16^ Another review analyzed the analgesic effectiveness of LB for abdominal fascial plane blocks, including laparoscopic, robotic, abdominal wall, and open surgery, and different techniques of block placement.^17^ Their evidence suggested similar analgesic effectiveness between LB and plain local anesthetics.

This review specifically aims to evaluate the effectiveness of ultrasound‐guided abdominal wall blocks for open abdominal surgery, focusing on their impact on opioid consumption and pain.

## METHODS

The study was registered on the PROSPERO website with registration number CRD42024527287. The full literature search can be found as supplemental information in Appendix S1.

### Literature search

The methods for data sources and study selection are described based on the Preferred reporting items for systematic reviews and meta‐analyses (Prisma) Checklist and the Prisma‐S extension to the PRISMA Statement for Reporting Literature Searches in Systematic Reviews.^18^, ^19^ A search strategy was developed by an experienced information specialist (EK) in cooperation with the lead author (MV). The search was developed in Embase.com, optimized for sensitivity and then translated to other databases.^20^ The search was carried out in the databases Medline ALL via Ovid (1946 to Daily Update), Embase.com (1971 till 11‐06‐2024), Web of Science Core Collection Science Citation Index Expanded (1975 till 11‐06‐2024); Social Sciences Citation Index (1975 till 11‐06‐2024); Arts and Humanities Citation Index (1975 till 11‐06‐2024); Conference Proceedings Citation Index‐Science (1990 till 11‐06‐2024); Conference Proceedings Citation Index – Social Science and Humanities (1990 till 11‐06‐2024) and Emerging Sources Citation Index (2015 till 11‐06‐2024) and the Cochrane Central Register of Controlled Trials via Wiley (1992 till 11‐06‐2024). Additionally, a search was performed in Google Scholar from which the 50 highest ranked references were downloaded using the software Publish or Perish.^21^ After the original search was performed in February 2023, the search was last updated on 11‐06‐2024 using the method described by Bramer et al.^22^

The search strategies for Medline and Embase include relevant thesaurus terms from Medical Subject Headings (MeSH) and Emtree terms, respectively. In all databases, terms were searched in titles, abstracts, and author keywords. The search contains terms for (1) wound infiltration or blocks and (2) abdominal surgery. Terms were combined with Boolean operators AND and OR, and proximity operators were used to combine terms into phrases. The full search strategies of all databases are available in the Appendix S1. No study registries were searched, but Cochrane CENTRAL retrieves the contents of ClinicalTrials.gov and the World Health Organization's International Clinical Trials Registry Platform. The references were imported into EndNote, and duplicates were removed using the method described by Bramer et al.^23^

### Inclusion criteria

All studies using LB in ultrasound‐guided abdominal wall blocks for open abdominal surgery, with reported outcomes of pain and/or opioid consumption, were taken into consideration. Only studies involving adult human patients (18 years or older) and the English language were included.

### Exclusion criteria

Studies including laparoscopic, robot‐assisted, abdominal wall, or non‐abdominal surgery were excluded. Surgeon‐placed, laparoscopic‐assisted, abdominal wall blocks using anatomical landmarks only or blocks under direct vision were also excluded. Studies comparing different abdominal wall blocks where both intervention and control groups used LB were excluded, as these studies essentially compare the effect of the block techniques rather than the specific effect of LB.

### Selection of studies

Two reviewers (MV and VB) independently screened titles and abstracts in EndNote using the method as described by Bramer et al.^24^ A third author (MD) was consulted to make a final decision in cases where the discussion was unresolved.

### Data extraction

Using a data extraction form created using a Microsoft Excel spreadsheet, the first two authors (MV and VB) independently extracted information regarding the author, year, country, study design, type of surgery, participants (number of participants, male, female), type of ultrasound‐guided block, control block, dosage and volume of injected anesthetic solutions, outcomes (pain and/or opioid consumption) and adverse events. Disagreements were resolved through discussion, and where necessary, a third author (MD) was consulted.

### Assessment of methodologic quality

The publications were categorized into randomized controlled trials (RCT), observational studies, or case reports. Risk of bias was assessed using version two of the Cochrane tool for randomized trials (RoB 2).^25^ The quality assessments were done by two independent reviewers (MV and VB) and discrepancies were discussed until consensus was reached. A third author (RJS) was consulted to make a final decision in cases where the discussion was unresolved.

## RESULTS

**FIGURE 1:**
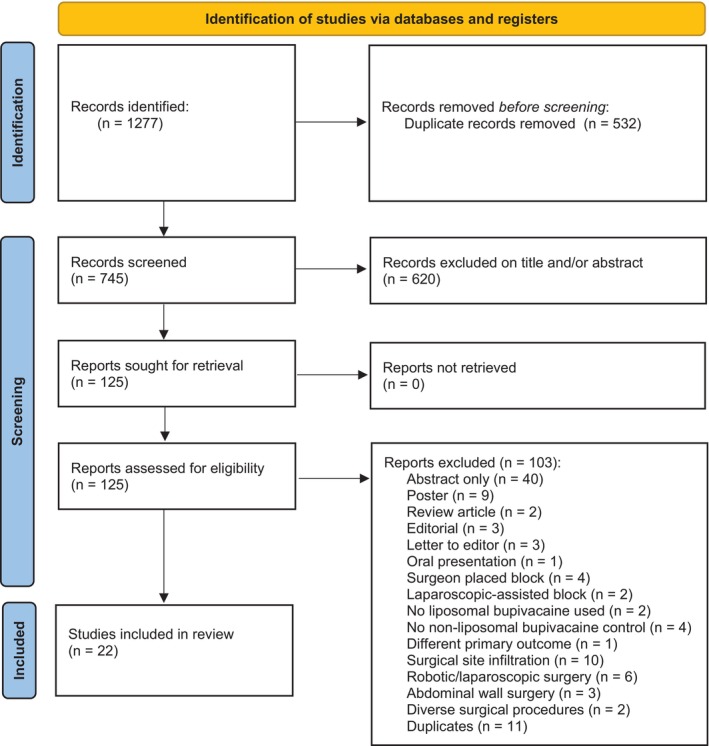
PRISMA flow diagram of inclusion of studies. *From:* Page et al.^26^

The literature search identified 1277 articles. After deduplication, 745 articles remained to be screened on title and abstract alone, of which 620 were excluded. The remaining 125 studies were then assessed for the availability of full text. Of these, 103 studies were excluded for various reasons, including duplicates (*n* = 11) that were missed during the deduplication procedure before screening. The flow chart of the inclusion of studies is reflected in Figure 1.

In 21 studies, 266 mg of LB was injected into the abdominal wall blocks, either undiluted (two studies) or diluted with bupivacaine and/or saline. One study did not describe the solution with LB that was used. Different volumes of anesthetic solution were used, ranging from 20 to 50 mL per injection. Four studies did not report the volume used.

A total of seven studies focused on cesarean delivery and abdominal hysterectomy, three studies investigated upper abdominal surgery, and 12 studies examined major abdominal surgery (all abdominal surgery excluding exclusively upper abdominal surgery, abdominal hysterectomy, and cesarean section). The study characteristics of all the included studies, including the primary outcome, results regarding opioid consumption and pain, and adverse events are reflected in Table 1. In the caesarean delivery and abdominal hysterectomy studies, TAP blocks, with or without intrathecal opioids, were investigated in all interventional groups. One study investigated subcostal TAP blocks without intrathecal opioids. Six studies investigated opioid consumption, and six reported on pain scores.

**TABLE 1:** Study characteristics of included studies reflecting the primary outcome, and results regarding opioid consumption, pain, and adverse events. Studies are subdivided into three categories: Cesarean section and hysterectomy, upper abdominal surgery, and major abdominal surgery.

Author, year, country	Study design	Intervention block with LB	Control	*n*	Type of surgery	Primary outcome	Outcome: Opioid consumption	Outcome: pain	Adverse events
*Caesarean delivery and abdominal hysterectomy*									
Habib et al.; 2021; USA	RCT	TAP ± ITM 50 μg ± ITF 15 μg. TAP solution: 266 mg LB for both groups. Volume not reported	ITM 150 μg ± ITF 15 μg	153	Cesarean delivery	Total opioid consumption through 72 hours or till hospital discharge	Significantly decreased total opioid consumption (including ITM and ITF) at 72 h in intervention groups. LB versus ITM: LSM [SE], 81.3 [5.3] vs. 120.2 [7.7] MME. LB + ITM versus ITM: LSM [SE], 87.2 [6.0] vs. 120.2 [7.7] MMEs. Only at 24 h did LB alone have increased post‐surgical opioid consumption	Overall pain scores statistically non‐inferior in all groups over 72 h. LSM [SE] LB 3.60 [0.21], LB + ITM 3.37 [0.22], ITM 3.84 [0.20] Significant reduction in incisional site pain scores in intervention groups at 72 h LSM (SE) LB 2.98 (0.22), LB + ITM 3.38 (0.22), ITM 4.02 (0.21)	Significantly less pruritis in both LB groups. Other opioid‐related side effects similar in all groups
Nedeljkovic et al.; 2020; USA	RCT	TAP + ITM 150 μg + ITF 15 μg. Solution: 50 mg bupivacaine +266 mg LB. 30 mL per injection. Total volume 60 mL	TAP + ITM 150 μg + ITF 15 μg. Solution: 50 mg bupivacaine. 30 mL per injection. Total volume 60 mL	186	Cesarean delivery	Total opioid consumption through 72 hours or till hospital discharge	Total postsurgical opioid consumption through 72 h reduced by 51.6% in intervention group. LSM [SE], 15.5 mg [6.67 mg] vs. 32.0 mg [6.25 mg] MED. Significant opioid reduction at 48 h and 7 days, but not at 14 days	Pain scores in the intervention group. Non‐inferior but not superior through 72 h	More PONV in LB group. More headache in bupivacaine group
Fidkowski et al.; 2021; USA	RCT	Subcostal TAP: Solution LB alone: 266 mg LB + 40 mL saline. Solution LB + bupivacaine: 100 mg bupivacaine +266 mg LB. For both groups: 30 mL per injection and total volume 60 mL	Subcostal TAP bupivacaine. Solution: 150 mg bupivacaine. 30 mL per injection. Total volume 60 mL	90	Abdominal hysterectomy	Time to first opioid analgesic and total opioid use 0–72 h post‐op	Median time to first opioid: [IQR] 51 [28–66] min in control group, 63 [44–102] min in LB alone group, and 51 [24–84] min in LB + bupivacaine group (*p* = 0.20)The median [IQR] total opioid consumption in the first 72 postoperative hours was 208 [155–270] mg in control group, 203 [153–283] mg in LB alone group, and 202 [116–325] mg in LB + bupivacaine group (*p* = 0.92)	First 24 h, all had NRS >5; no difference in maximum and minimum NRS in all time intervals up to 72 h	None
Baker et al.; 2018; USA	Observational study	TAP LB 266 mg + ITM 100 μg. Solution not reported	ITM 100 μg	201	Cesarean delivery	Pain scores and opioid consumption up to POD 3	Significant reduction in opioid consumption in LB group. Mean MED POD 1: LB group 25 mg vs. 60 mg in control group; POD 2: LB group 25.4 mg vs. 51.8 mg in control group; POD 3: LB group 42.0 mg vs. 64.1 mg in control group	Significant reduction in pain scores LB group. Total AUC of NRS pain scores from 0 to 3 days: LB group 132.8 (98.3) vs. control group 246.3 (102.8)	Significant decrease in opioid‐related side effects in LB group
Feierman et al.; 2021; USA	Observational study	TAP + spinal: ITM 100 μg + ITF 15 μg OR TAP + epidural: 25 mg lidocaine + morphine 1 mg. TAP solution: 75 mg bupivacaine + 266 mg LB. 25 mL per injection. Total volume 50 mL	Spinal: ITM 100 μg + ITF 15 μg +/− TAP OR Epidural 25 mg lidocaine + morphine 1 mg +/− TAP. TAP solution: Bupivacaine 0.25%. Volume not reported	206	Cesarean delivery	Opioid consumption at fixed intervals till >72 h postoperatively and length of stay	Significant decrease in the number of patients using postoperative opioids: 54%–60% control group decreased to 18% (*p* < 0.001) in the LB TAP + neuraxial morphine groupMME's not reported	Not reported	None
Alexander et al.; 2022; USA	Observational study	TAP. Solution: 50 mg bupivacaine +266 mg LB. 20 mL per injection. Total volume 40 mL	TAP. Solution: Ropivacaine 0.5%. 20 mL per injection. Total volume 40 mL	215	Abdominal hysterectomy	Inpatient opioid consumption at fixed intervals up to 48 h	Decreased opioid consumption at 24 h reaching significance at 48 h (LB TAP mean MEQ [SD] 27.9 [±26.5] versus control mean MEQ [SD] 37.4 [±34.2] Significant decrease in total opioid consumption in LB group. LB TAP mean MEQ [SD] 60.2 [±40.9] versus control mean MEQ [SD] 78.7 [±61.2]	No significant differences between groups at all time intervals. Mean pain scores at 48 h [SD]: LB TAP 2.8 [±3.1] versus control 3.6 [±3.9]	Incidence of nausea not significantly different
Feierman et al.; 2017; USA	Case series	ITF 15 μg + ITM 200 μg + TAP. TAP solution: 75 mg bupivacaine +266 mg LB. 25 mL per injection. Total volume 50 mL	Not applicable	12	Cesarean delivery	Pain scores at fixed intervals up to 72 h or discharge (if earlier)	Only three patients received opioids	Low mean pain scores (±SD) at all time intervals. 1.0 ± 1.4, 1.4 ± 2.1, 1.7 ± 1.9, 1.9 ± 3.3 and 1.9 ± 2.3 at 6, 12, 24, 48, and 72 h respectively	None
*Upper abdominal surgery*									
Aiken et al.; 2022; USA	Observational study	QL (*n* = 13)/TAP (*n* = 34). Solution: 75 mg bupivacaine +266 mg LB. 25 mL per injection. Total volume 50 mL	TEA	102	Oncology with epigastric incision	Inpatient opioid consumption, severe postoperative pain	QL and TAP groups significantly more opioid consumption than TEA: 0–24 h (116 vs. 94 MME), 24–48 h (94 vs. 23 MME) and entire hospital stay (377 vs. 122 MME)	No significant difference between block groups and TEA in reported severe pain at 0–24 h (76.7% vs. 60.8%, 24–48 h (18.5% vs. 11.1%) and entire hospital admission (70.2% vs. 67.2%)	No significant differences between groups regarding hypotension, frequency of ileus or urinary retention
Amundson et al.; 2018; USA	Observational study	5‐point TAP. Solution: 75 mg bupivacaine +266 mg LB. 10 mL per injection. Total volume 50 mLIntrathecal hydromorphone 100–150 μg	Intrathecal hydromorphone 100–150 μg	77	Living liver donation	NRS POD 0 and 1	Block group significant decrease in median [IQR] opioid consumption MME. PACU: 0 [0–10] vs. 9 [1–25]. POD 0: 7 [0–15] vs. 18 [5–36]. No significant decrease thereafter	POD 0 pain scores significantly better in block group than control group (2.4 vs. 3.5). Not significantly different thereafter	PONV similar in both groups
Yeap et al.; 2019; USA	Observational study	TAP: Solution with LB not reported	TAP ropivacaine 0.2% (bolus 30 mL per side) + catheters or intravenous opioid alone	197	Pancreas transplant	Pain scores and total opioid consumption POD 1–5.	Opioid consumption lower for both TAP groups in comparison to intravenous opioid group POD 1–5 Median (range) opioid usage (MME) in PACU highest for TAP catheter group 12 (0–124), and significantly lower for IV opioid 0 (0–80) and LB groups 8 (0–60)Significantly lower total median (range) opioid consumption MME POD 1–5 for block groups: Lowest for LB group 349 (65–1655), TAP catheter group 484 (36–3064) and IV opioid group 529 (102–7354)	Median pain scores in PACU significantly lower for IV opioid only group compared with both TAP groups (2.0 vs. 5.6 and 4.6). Also true for median average daily postoperative pain scores POD 1–5 (2.6 vs. 4.1 and 3.4)LB group significantly lower pain scores than Ropivacaine catheter group POD 1–5: median (range) NRS 3.4 (0–7) vs. 4.1 (0–7)	No significant difference in PONV or other opioid‐related side effects between all groups
*Major abdominal surgery*									
Cata et al.; 2021; USA	RCT	Four quadrant TAP^†^. Solution: 150 mg bupivacaine +266 mg LB. 20 mL per injection. Total volume 80 mL	TEA (if appropriate bolus 300–800 μg hydromorphone pre‐incision)—in total 60% median bolus 800 μg)	68	CRS‐HIPEC	Quality of Recovery (QoR) POD 2	TAP group significantly higher opioid consumption without significantly increased opioid‐related adverse events at 48 h Median MME (range) on POD 2 TAP group vs. TEA: 21 (1.8–104.8) vs. 4.5 (0–51)	Using QoR15 questionnaire: 4 quadrant TAP non‐inferior to TEA up to POD 2, possibly up to POD 30 Median Verbal NRS at rest and at cough TAP vs. TEA not significantly different at POD 2 (3 vs. 3 *p* = 0.86; 6 vs. 6, *p* = 0.97)	More hypotension in TEA group. Opioid‐related side effects, ileus and PONV similar in both groups
Turan et al.; 2022; USA	RCT	Preoperative bilateral or four‐quadrant TAP. Solution: 100 mg bupivacaine +266 mg LB + saline. 40 mL per injection for bilateral TAP, 20 mL per injection for four‐quadrant TAP. Total volume 80 mL	TEA	498	Major abdominal surgery >74% open procedures	Average pain scores at rest and total opioid consumption during 72 h	TAP group significantly more opioid over 72 h—Median [IQR]: 56 [20–122] vs. 35 [13–94] without significant mean difference in opioid‐related side effects	No significant differences in mean pain scores (SD) over 72 h (TAP 4.3 (1.8) vs. TEA 4.2 (1.8) 90 day follow‐up not reported	More hypotension in TEA group. Similar opioid‐related side effects in both groups
Ayad et al.; 2016; USA	Observational study	TAP preoperative. Solution: 100 mg bupivacaine +266 mg LB. 20 mL per injection. Total volume 40 mL	Epidural or intravenous PCA (hydromorphone, fentanyl or morphine)	318	Major lower abdominal surgery (96% open)	Average pain scores and total MME at 72 h	Median total MME [IQR] TAP 88 [28–181] vs. epidural 137 [82–246] vs. PCA 78 [36–184]. TAP and EDA not preferable to PCA regarding opioid requirement	Average NRS (SD) until 72 h TAP 4.0 ± 1.7 vs. epidural 4.3 ± 1.8 vs. PCA 3.8 ± 2.0	No significant difference in incidence of postoperative paralytic ileus
Stokes et al.; 2017; USA	Observational study	TAP post‐induction. Solution: 25 mg bupivacaine +266 mg LB + 20 mL saline. 25 mL per injection. Total volume 50 mL	TAP >90% post‐induction. Solution: 100 mg bupivacaine. 20 mL per injection. Total volume 40 mL	407	Colorectal surgery— > 50% open	Inpatient pain scores, analgesic and opioid consumption	Similar cumulative opioid consumption (MME) in both groups (open surgery population) 64.4 vs. 65.6 *p* = 0.93	No subset analysis of pain scores for open surgery population	No significant differences in surgical complications. One patient with necrotizing soft tissue infection at TAP puncture site
Dammann et al. 2020; USA	Observational study	TAP. Solution: LB + 0,25% bupivacaine + saline. Volumes and dosages not reported	No block	52	Major abdominal surgery	Pain scores and opioid consumption POD 0–5	Reduction in opioid consumption from POD 2–5 in LB group.	Significantly less patients with severe pain in LB group	Significant reduction in adverse events in LB group
Osuchukwu et al.; 2024; USA	Observational study	TAP. Solution: 50 mg bupivacaine +266 mg LB. 30 mL per injection. Total volume 60 mL	TAP. Solution: 120 mg bupivacaine. 30 mL per injection. Total volume 60 mL	178	Lower abdominopelvic surgery	Total opioid consumption through 72 h postoperatively	Median MME [IQR] LB group 47.5 [18–91.8] vs. non‐LB group 88 [43.8–160] *p* = 0.045. No significance after adjustment for covariates *p* = 0.11	No difference in average pain intensities between groups (intervention vs. control) over time (POD 0, 4.7 ± 2.4 vs. 5.2 ± 1.9; POD 1 4.5 ± 2.2 vs. 4.6 ± 2.2; POD 2 3.9 ± 2.3 vs. 4.0 ± 2.1, respectively) either before (*p* = 0.34) or after (*p* = 0.72) adjustment for covariates	Not reported
Boyev et al. 2024; USA	Observational study	Subcostal TAP, QL and/or rectus sheath blocks. Solution LB 166 mg + bupivacaine 0.25%. Volume not reported	Subcostal TAP, QL and/or rectus sheath blocks. Bupivacaine 0.25% or ropivacaine ± dexamethasone, ±dexmedetomidine. Volume not reported	219	Major abdominal surgery	Total MME > 150 mg and proportion of patients with severe pain over the first 0–72 h	Similar number of patients who reached >150 mg MME over the first 0–72 h: LB (19.9%, 29/146) and control (16.4%, 12/73, *p* = 0.586)	Severe pain experienced similar in both groups: LB 44% (64/146) and control 53% (39/73) *p* = 0.198	Not reported
Elsharkawy et al.; 2020; USA	Observational study	Anterior subcostal QL (*n* = 3)/TAP (*n* = 7) continuous infusion ropivacaine. LB injected before catheter removal. LB solution unilateral: 266 mg + saline. Total volume 30 mL. LB solution bilateral: LB 266 mg + 20 mL saline. 20 mL per injection. Total volume 40 mL	No block	10	Major abdominal surgery	Pain tolerance 48 h after catheter removal and LB injection (median LB injection on POD 3)	Not reported	Pain scores decreased from 1 h after LB injection and catheter removal up to 48 h after LB injection	None
Patzkawski et al.; 2015; USA	Case report	Rectus Sheath. Solution: LB 266 mg + saline 60 mL. 40 mL per injection. Total volume 80 mL	Not applicable	1	Laparoscopic appendectomy converted to open right hemicolectomy	Observation of opioid consumption and pain scores	MME up to 72 h: 198.6. Thereafter increased opioid consumption attributed to intra‐abdominal abscess formation	30 pain scores in 72 h: NRS between 2 and 3	Intra‐abdominal abscess formation
Elsharkawy et al.; 2016; USA	Case report	QL on left side. Solution: 133 mg LB + saline. Total volume 25 mL	TAP on right side Solution: LB 133 mg + saline. Total volume 25 mL	1	Subtotal colectomy	Sensory level and analgesia achieved	190 mg intravenous MME over 72 h	Verbal NRS 2–4 over 72 h	None
Arsky Lombardi et al.; 2023; USA	Case report	Rectus sheath and TAP. Solution: 75 mg bupivacaine +266 mg LB. 12.5 mL per injection. Total volume 50 mL	TEA POD 1 due to severe persistent pain despite additional IV opioids and ketamine	1	Transverse colectomy and right‐sided colostomy in patient with intrathecal morphine pump	Pain management, local anesthetic systemic toxicity	Continuous intrathecal morphine, PCEA, hydromorphone PCA and continuous infusion ketamine	Moderate to mild pain after addition of TEA other analgesic modalities.	None
Vereen et al.; 2024; The Netherlands	Case report	Rectus Sheath. Solution: 100 mg bupivacaine + LB 266 mg + saline 40 mL. 50 mL per injection. Total volume 100 mL	Not applicable	3	Open oncologic abdominal surgery	Observation of opioid consumption and pain scores up to POD 3 and 2 weeks postoperatively	Mean MME low at POD 3 (mean 15) and zero at 2 weeks	Mean NRS POD 3 3.67 and 0.67 at 2 weeks	None

Abbreviations: AUC, area under the curve; CRS‐HIPEC, cytoreductive surgery and hyperthermic intraperitoneal chemotherapy; IQR, interquartile range; ITF, intrathecal fentanyl; ITM, intrathecal morphine; IV, intravenous; LB, liposomal bupivacaine; LSM [SE], least squares mean [standard error]; MED, oral morphine equivalent dosing; MEQ, milligrams of morphine equivalent; MME, milligram morphine equivalent; NRS, numeric rating scale; PACU, post‐anesthesia care unit; PCA, patient controlled analgesia; PCEA, patient‐controlled epidural analgesia; POD, postoperative day; PONV, postoperative nausea and vomiting; QL, quadratus lumborum; SD, standard deviation; TAP, transversus abdominis plane; TEA, thoracic epidural anesthesia.

In the three upper abdominal surgery studies, conventional TAP, 5‐point TAP, and QL blocks were investigated in the interventional groups. The control groups were either TEA, intrathecal hydromorphone, single shot or continuous TAP, or intravenous opioids. All three studies reported on opioid consumption and pain scores.

In the major abdominal surgery group, interventional groups received LB in the form of bilateral or 4‐quadrant TAP blocks (anterior subcostal), QL blocks, or rectus sheath blocks. Eleven studies reported on both opioid consumption and pain scores, while one study only reported on pain scores.

Types of surgeries, as well as interventional blocks, were diverse. TAP blocks were investigated the most; however, the timing and approach of TAP block placement were not uniform. The use of intrathecal opioids was also not standard in all studies. These factors prevented the pooling of results for a meta‐analysis.

**FIGURE 2:**
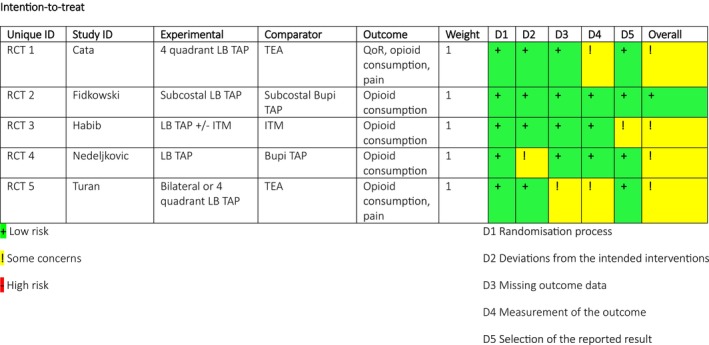
Results of the Cochrane risk‐of‐bias tool (RoB 2) assessment.

Using the RoB 2 tool for RCTs, one study had a low risk of overall bias, and four studies had some concerns, as seen in Figure 2. Three studies had some concerns in one domain, and one study had concerns in two domains. The impact of bias on the overall outcome was interpreted to be low.

### Outcomes: Opioid consumption and pain scores

#### Randomized controlled trials

A total of five RCTs were analyzed. Of these, two reported on cesarean delivery, one on abdominal hysterectomy, and two on major abdominal surgery. In the two studies investigating patients undergoing cesarean delivery with neuraxial anesthesia, using different solutions of local anesthetic in TAP blocks, with or without intrathecal morphine (ITM), opioid consumption was reduced with corresponding non‐inferior overall pain scores in patients receiving LB TAP at 72 h.^27^, ^28^ One study showed no benefit of subcostal LB TAP blocks in patients undergoing abdominal hysterectomy.^29^

Cata et al.^30^ showed a significantly higher opioid consumption on postoperative day (POD) 2 in the LB TAP group versus TEA with median MME (morphine milligram equivalents) (range) 21 (1.8–104.8) versus TEA 4.5 (0–51) *p* < 0.001, with comparable pain scores (median verbal NRS at rest 3 versus 3 *p* = 0.86, and at cough 6 vs. 6, *p* = 0.97), for patients undergoing CRS‐HIPEC. Similar results were reported in a larger study comparing bilateral or four‐quadrant LB TAP with TEA in major abdominal surgery over 72 h.^31^

#### Observational studies

In total, 12 observational studies were included: three in the cesarean delivery and abdominal hysterectomy group, three in the upper abdominal surgery group, and 6 in the major abdominal surgery group.

Two studies demonstrated that patients undergoing cesarean section with neuraxial anesthesia had decreased opioid consumption in the TAP groups.^32^, ^33^ Pain scores in one of these studies were also decreased.^32^ Most patients in these studies received ITM, which was included in the analyses of total opioid consumption in each study. One study demonstrated a significant decrease in opioid consumption and similar pain scores in the LB TAP group for patients undergoing abdominal hysterectomy.^34^

Three studies analyzed patients undergoing surgery in the upper abdomen, comparing LB TAP blocks to various control groups, whereby varied outcomes were demonstrated.^35^, ^36^, ^37^ An additional finding in two studies was an earlier return of bowel function in the LB TAP groups, as well as in all TAP groups when compared with IV opioids alone.^36^, ^37^

Results for major abdominal surgery showed considerable variation. One study demonstrated that LB TAP was non‐inferior to TEA regarding pain and opioid consumption.^38^ Stokes et al. showed similar opioid consumption for both TAP groups in the subset analysis of patients undergoing open surgeries; however, no pain scores were reported for these patients.^39^ One study comparing LB TAP to no block reported decreased opioid consumption from PODs two to five and fewer patients in the LB group having severe pain.^40^ Two other studies found similar opioid consumption and pain scores in both the LB and control groups.^41^, ^42^ One study demonstrated decreased pain scores after the injection of LB into TAP and QL catheters, which were postoperatively infused with ropivacaine just prior to catheter removal on (median) POD 3.^43^

#### Case reports

One case series and four case reports were analyzed. The case series (*n* = 12) of LB TAP and intrathecal opioids after cesarean section described low mean pain scores at different time intervals up to 72 h postoperatively, as well as low opioid usage.^44^ One case report described the use of LB in RS blocks in a laparoscopic appendectomy converted to an open right hemicolectomy.^45^ The patient had mild postoperative pain scores facilitating ambulation on the day of surgery. Another case report described the sensory level and analgesia obtained with LB in both a left‐sided QL block compared with a right‐sided TAP block in a patient undergoing a subtotal colectomy.^46^ The patient received opioids for the pain he experienced more on the side of the TAP block. One case report described TEA placement with successful analgesia alongside intravenous opioids and ketamine after ineffective analgesia with preoperatively placed abdominal wall blocks with LB in a patient with an ITM pump for chronic pain after transverse colectomy.^47^ Another case report described three patients with good analgesia and low pain scores after RS blocks after open oncological surgery.^48^

## DISCUSSION

This literature search was conducted to review the effectiveness of LB specifically in ultrasound‐guided abdominal wall blocks, in open abdominal surgery, with a focus on opioid consumption and pain. All included studies administered 266 mg of LB, either undiluted or diluted with bupivacaine and/or saline. No adverse events directly related to LB were reported.

In the included studies, patients undergoing cesarean section with neuraxial anesthesia clearly demonstrated a positive effect of LB TAP over ITM groups alone, as reflected by decreased opioid consumption and decreased or non‐inferior pain scores. Furthermore, they also demonstrated a decreased length of stay.^27^, ^28^, ^32^, ^33^, ^44^ As nearly all patients received intrathecal opioids, it would be interesting to have data about the effect of the LB TAP block alone. The benefit of the LB TAP in the direct postoperative period was clear; however, data beyond 72 h are lacking. This is an important factor to consider, as the incidence of chronic postsurgical pain following cesarean section is 25%.^49^

From this data, no definite conclusions can be drawn about the efficacy of LB in ultrasound‐guided abdominal wall blocks when considering all types of open abdominal surgeries as one entity. Earlier return of bowel function is a beneficial factor to be considered, as was demonstrated in the LB TAP groups in the observational studies of surgery in the upper abdomen.^36^, ^37^ Although LB TAP was non‐inferior to TEA regarding pain in the two RCTs analyzed for major abdominal surgery, the conflicting results regarding opioid consumption make it difficult to draw clear conclusions.^30^, ^31^ Opioids are necessary in combination with TAP blocks as these do not manage visceral pain. One could thus argue in favor of LB TAP, as there were similar results in all groups and limited opioid‐related adverse effects. Further research looking at the long‐term effects of pain and opioid consumption might help us to decide in this regard.

Ultrasound‐guided TAP blocks anesthetize the anterolateral abdominal wall when local anesthetic is deposited into the fascial plane between the transversus abdominis muscle and the internal oblique muscle.^10^, ^13^ Theoretically, dermatomes T7–T12 can be covered by using this block; however, various factors such as the approach to the TAP, volume injected, type of local anesthetic used, and anatomical variations can lead to a variable block.^10^ With a few exceptions, all the articles in this review concerned TAP blocks for different types of surgical incisions. Additionally, the TAP blocks were performed using various approaches, such as the four‐quadrant TAP or subcostal TAP. However, the posterior approach to the TAP could anesthetize the lateral cutaneous branches of the main segmental thoracolumbar nerves and thereby improve analgesia of the lateral and paramedian parts of the abdominal wall.^10^ This effect could be missed if the needle tip is placed ventral to the origin of the lateral cutaneous branches and may have an impact on analgesia and opioid consumption.

The importance of correct needle placement in combination with correct local anesthetic dosage and adherence to a multimodal postoperative analgesia regime for the efficacy of the LB TAP block was demonstrated in one study.^28^ Patients analyzed in their RCT showed no benefit of LB TAP when the block was performed inaccurately. One study described the use of ultrasound‐guided subcostal TAP blocks for abdominal hysterectomy with a midline incision.^29^ As the subcostal TAP block covers the anterior cutaneous branches of T6–T9, it could be questioned if the negative outcome of this study was to be expected, as the TAP approach may have been too cranial for effective analgesia with lower abdominal midline incisions. Furthermore, a posterior approach to the TAP, or RS blocks, could have provided better analgesia for this type of incision.

Ultrasound‐guided RS blocks are indicated for umbilical surgery or midline laparotomy.^50^ Needle placement and injection of local anesthetic at the lateral part of the rectus abdominis muscle just below the muscle and ventral to the posterior RS provide analgesia in the midline from T7 to T12. One case report described the use of ultrasound‐guided RS blocks for midline laparotomy with good outcomes regarding pain, mobilization, and return of bowel function.^48^ A recent quality improvement study reported decreased workload for nursing staff without compromising analgesia after implementation of RS blocks with LB instead of using RS catheters.^51^ The volume needed for optimal dermatomal spread after RS block has not yet been extensively researched. One study described the spread and efficacy of a fixed volume (20 mL) of levobupivacaine 0.375% for umbilical hernia repair surgery, with 97% of patients having excellent postoperative analgesia.^52^ A recent study of seven cadavers suggests that at least 30 mL per side is needed for the spread from xiphoid to pubis.^53^

None of the included studies reported on long‐term results. Considering the evidence from the current available literature, it is unknown if LB could have a possible impact on long‐term outcomes. Future research aimed at long‐term data, up to 90 days, on opioid consumption and/or the prevalence of chronic postsurgical pain with the use of LB in ultrasound‐guided abdominal wall blocks might be the key to making decisive decisions. With the declining use of epidural analgesia and the challenge of limiting opioid consumption, having conclusive data about the efficacy of LB in abdominal wall blocks would be valuable. One case report has demonstrated that the QL block may be more effective than the TAP block.^46^ Further data on the efficacy of QL and erector spinae plane blocks, where the visceral component of pain could also be managed, is an interesting topic for further research. Insight into the efficacy of LB in ultrasound‐guided abdominal wall blocks as part of a multimodal analgesic regime might prioritize its use if the incidence of chronic postsurgical pain and prolonged opioid use with the risk of dependency is decreased as well.

There are limitations to this review. The search strategy has been limited to adults and articles published in the English language. Unlike the systematic reviews of Zhu et al. and Hussain et al., only papers describing the use of ultrasound‐guided regional techniques in open abdominal surgery were included.^16^, ^17^ This was mainly due to the current practice at our hospital regarding the placement of abdominal wall blocks in open abdominal surgery, as well as the fact that ultrasound‐guided placement of locoregional techniques has become the gold standard.^54^ The registration of LB, currently limited to the United States of America since 2011, the European Union since 2020, and the United Kingdom since 2021, could explain why all the included studies, except one case report, are from the USA. The limited data on the use of ultrasound‐guided RS blocks with LB for open abdominal surgery could be attributed to the fact that LB was first registered for use in shoulder and knee surgery, and from 2015 for field blocks.^55^ Data on the effect of LB in QL and erector spinae plane blocks is lacking, possibly because these blocks have not yet been widely adopted for pain management in open abdominal surgery. The heterogeneity of the data prevented the possibility of a meta‐analysis, thereby precluding clear recommendations based on effectiveness. Finally, costs and sustainability are current issues beyond the scope of this review but are important factors to consider.

From this review, it can be concluded that patients who undergo cesarean sections with neuraxial anesthesia may benefit from ultrasound‐guided LB TAP combined with ITM in the immediate postoperative period, as it results in decreased pain and opioid consumption. However, the evidence for the effectiveness of LB TAP in other open abdominal surgeries is inconclusive. The evidence from this review suggests that abdominal wall blocks with LB could be a viable alternative when epidural analgesia is not preferred or feasible. Further research, including longer‐term outcomes regarding the use of LB in ultrasound‐guided abdominal wall blocks for open abdominal surgery, is warranted.

## AUTHOR CONTRIBUTIONS

This study was designed by M.V., M.D., E.K., and V.B., M.V., and V.B. analyzed the data, and the results were critically examined by all authors. M.V. had a primary role in preparing the manuscript, which M.D., S.H., and R.J.S. edited. All authors reviewed and approved the final version of the manuscript and agree to be accountable for all aspects of the work.

## FUNDING INFORMATION

None.

## CONFLICT OF INTEREST STATEMENT

None.

## PATIENT CONSENT

No patient consent was required for this systematic review.

## REVIEW REGISTRATION

This study has been registered on the PROSPERO website with registration number CRD42024527287.

## Supporting information


Appendix S1.

## Data Availability

The data that supports the findings of this study are available in the Appendix S1 of this article.
