# Supplementary material for: The effectiveness of liposomal bupivacaine in ultrasound‐guided abdominal wall blocks after open abdominal surgery: A systematic review
Source: Pain Pract. 2025 Feb 19;25(3):e70016. doi: 10.1111/papr.70016 (PMC11837461; doi:10.1111/papr.70016)
Supplement: Supplementary file 1 — Appendix S1. [file PAPR-25-0-s001.docx]

**Appendix 1. Literature search strategy and search strings**

| **Database searched** | **Platform** | **Years of coverage** | **Records** | **Records after duplicates removed** |
| --- | --- | --- | --- | --- |
| Medline ALL | Ovid | 1946 - Present | 185 | 184 |
| Embase | Embase.com | 1971 - Present | 513 | 334 |
| Web of Science Core Collection* | Web of Knowledge | 1975 - Present | 267 | 76 |
| Cochrane Central Register of Controlled Trials | Wiley | 1992 - Present | 212 | 104 |
| Additional Search Engines: Google Scholar** (100 top ranked) | | | 100 | 47 |
| **Total** | | | **1277** | **745** |

*Science Citation Index Expanded (1975-present) ; Social Sciences Citation Index (1975-present) ; Arts & Humanities Citation Index (1975-present) ; Conference Proceedings Citation Index- Science (1990-present) ; Conference Proceedings Citation Index- Social Science & Humanities (1990-present) ; Emerging Sources Citation Index (2005-present)

**Google Scholar was searched via "Publish or Perish" to download the results in EndNote.

No other database limits were used than those specified in the search strategies

**Inclusion criteria:**

Liposomal bupivacaine in ultrasound-guided abdominal wall blocks

Open abdominal surgery

Reported outcome of pain and/or opioid consumption

Adult human patients (18 years or older)

English language were included.

**Exclusion criteria:**

Laparoscopic, robot-assisted, abdominal wall or non-abdominal surgery.

Surgeon-placed, laparoscopic-assisted or abdominal wall blocks using anatomical landmarks only

Blocks under direct vision

Intervention and control groups both using liposomal bupivacaine.

**Embase**

('liposomal delivery'/de OR (Bupivacaine/de AND liposome/exp) OR (liposom* OR exparel* OR ((lipid*) NEAR/6 (bupivac*))):ab,ti,kw) **AND** ('transversus abdominis plane block'/de OR 'erector spinae plane block'/de OR ‘nerve block’/exp OR ‘erector spinae muscle’/de OR ‘abdominal wall musculature’/exp OR Bupivacaine/de OR (block* OR ((fasc*) NEAR/6 (plane)) OR transversus-abdominis-plane* OR TAP OR rectus-sheath* OR eractor-spinae* OR quadratus-lumborum* OR infiltration* OR Bupivacain*):ab,ti,kw) **AND** (‘abdominal surgery’/exp OR Abdomen/exp OR ‘cesarean section’/exp OR ‘uterus surgery’/exp OR (abdom* OR digestive-system* OR laparotom* OR ((biliary-tract* OR gastrointest* OR gastro-intest* OR liver OR spleen OR hepat* OR colo* OR uter*) NEAR/3 (surg* OR transplant* OR operat*)) OR colorectom* OR gastrectom* OR cesarean* OR caesarean* OR colectom*):ab,ti,kw) NOT ([animals]/lim NOT [humans]/lim) AND [ENGLISH]/lim

**Medline**

((Bupivacaine/ AND Liposomes/) OR (liposom* OR exparel* OR ((lipid*) ADJ6 (bupivac*))).ab,ti,kf.) **AND** (exp Nerve Block/ OR Bupivacaine/ OR (block* OR ((fasc*) ADJ6 (plane)) OR transversus-abdominis-plane* OR TAP OR rectus-sheath* OR eractor-spinae* OR quadratus-lumborum* OR infiltration* OR Bupivacain*).ab,ti,kf.) **AND** (exp Abdomen/ OR exp Digestive System Surgical Procedures/ OR Cesarean Section/ OR exp Obstetric Surgical Procedures/ OR (abdom* OR digestive-system* OR laparotom* OR ((biliary-tract* OR gastrointest* OR gastro-intest* OR liver OR spleen OR hepat* OR colo* OR uter*) ADJ3 (surg* OR transplant* OR operat*)) OR colorectom* OR gastrectom* OR cesarean* OR caesarean* OR colectom*).ab,ti,kf.) NOT (exp Animals/ NOT Humans/) AND english.la.

**Cochrane**

((liposom* OR exparel* OR ((lipid*) NEAR/6 (bupivac*))):ab,ti,kw) **AND** ((block* OR ((fasc*) NEAR/6 (plane)) OR transversus NEXT abdominis NEXT plane* OR TAP OR rectus NEXT sheath* OR eractor NEXT spinae* OR quadratus NEXT lumborum* OR infiltration* OR Bupivacain*):ab,ti,kw) **AND** ((abdom* OR digestive NEXT system* OR laparotom* OR ((biliary NEXT tract* OR gastrointest* OR gastro NEXT intest* OR liver OR spleen OR hepat* OR colo* OR uter*) NEAR/3 (surg* OR transplant* OR operat*)) OR colorectom* OR gastrectom* OR cesarean* OR caesarean* OR colectom*):ab,ti,kw)

**Web of Science**

TS=(((liposom* OR exparel* OR ((lipid*) NEAR/5 (bupivac*)))) **AND** ((block* OR ((fasc*) NEAR/5 (plane)) OR transversus-abdominis-plane* OR TAP OR rectus-sheath* OR eractor-spinae* OR quadratus-lumborum* OR infiltration* OR Bupivacain*)) **AND** ((abdom* OR digestive-system* OR laparotom* OR ((biliary-tract* OR gastrointest* OR gastro-intest* OR liver OR spleen OR hepat* OR colo* OR uter*) NEAR/2 (surg* OR transplant* OR operat*)) OR colorectom* OR gastrectom* OR cesarean* OR caesarean* OR colectom*)) NOT ((animal* OR rat OR rats OR mouse OR mice OR murine OR dog OR dogs OR canine OR cat OR cats OR feline OR rabbit OR cow OR cows OR bovine OR rodent* OR sheep OR ovine OR pig OR swine OR porcine OR veterinar* OR chick* OR zebrafish* OR baboon* OR nonhuman* OR primate* OR cattle* OR goose OR geese OR duck OR macaque* OR avian* OR bird* OR fish*) NOT (human* OR patient* OR women OR woman OR men OR man))) AND LA=(English)

**Google Scholar**

liposomal|exparel “abdominal|abdominis|abdominus|TAP|ESP|erector block|blocks”

liposomal|exparel 'abdominal|abdominis|abdominus|TAP|ESP|erector block|blocks'
